# Supplementary material for: Self-care needs among international migrants and travellers: A systematic review and meta-synthesis
Source: PLoS One. 2026 Mar 10;21(3):e0344437. doi: 10.1371/journal.pone.0344437 (PMC12974874; doi:10.1371/journal.pone.0344437)
Supplement: S1 Appendix — (DOCX) [file pone.0344437.s001.docx]

**S1 Appendix. The Enhancing Transparency in Reporting the Synthesis of Qualitative Research (ENTREQ) Statement [17]**

| **No** | **Item** | **Description** | **Page Number** |
| --- | --- | --- | --- |
| 1. | Aim | The aim of this review was to determine the self-care needs of international travellers and temporary migrants, and to assess how these needs align with existing self-care frameworks. | 2, 5 |
| 2. | Synthesis methodology | Thematic synthesis | 2, 8 |
| 3. | Approach to searching | The pre-planned approach with comprehensive search strategies was used to seek all available studies. | 6-7 |
| 4. | Inclusion criteria | International tourists, backpackers, international students, business travellers and transient or seasonal migrant workers were included. Literature must be qualitative, multi-methods or mixed-methods primary research articles focusing on self-care among international travellers and migrants. All qualitative research designs were considered. The language and publication date were not restricted. | 6 |
| 5. | Data sources | Information sources were MEDLINE, Embase, International Pharmaceutical Abstracts, and PsycINFO databases via Ovid, and CINAHL via EBSCO. Searches were performed on 5 October 2022 and updated on 30 October 2024. | 6-7 |
| 6. | Electronic search strategy | The concepts of self-care and travel were elaborated into keywords and search strings depending on the database (S3 Appendix). | 7, S3 Appendix |
| 7. | Study screening methods | Researchers ANWP and JC independently screened the title, abstract, and full text. Any discrepancies were discussed with Researcher CS until a consensus was reached. | 7 |
| 8. | Study characteristics | Characteristics of included studies were the year of publication, aim(s), study location, sample, sample size, data collection method, and data analysis (Table 1). | 12 |
| 9. | Study selection results | The searches identified 3275 citations. Upon deduplication, abstract screening, and assessment of eligibility criteria, 17 studies were retained for inclusion. The details of the screening process and reasons for exclusion were presented in Figure 1. | 10-11 |
| 10. | Rationale for appraisal | Appraisal was based on methodological rigour to assess aspects such as sampling strategy, data collection and analysis methods, the researcher’s position, the general sense of the results, conclusion-making, and transferability (S4 Appendix). | 7 |
| 11. | Appraisal items | The Center for Evidence-Based Medicine’s Critical Appraisal of Qualitative Studies sheet was used to appraise the included studies. | 7 |
| 12. | Appraisal process | Researchers ANWP and JC appraised 17 studies independently by answering “Yes”, “No”, or “Unclear” for every criterion. Discrepancies were resolved by consensus between Researchers ANWP, JC, and CS. | 7 |
| 13. | Appraisal results | All studies were considered acceptable because most questions were answered “Yes”. Most studies did not report the researcher’s position owing to variations in reporting standards between journals. The appraisal results were presented in S4 Appendix. | S4 Appendix |
| 14. | Data extraction | All texts under the “Results” or “Findings” section were considered data. | 8 |
| 15. | Software | Covidence for screening and quality appraisal and NVivo 13 for data analysis | 7, 8 |
| 16. | Number of reviewers | Two reviewers (ANWP and CS) | 7, 8 |
| 17. | Coding | Researcher ANWP performed a line-by-line coding in close consultation with Researcher CS. The themes generated by the study’s authors were used to name the codes in most instances. | 8 |
| 18. | Study comparison | All included studies were read before data extraction. Each study was extracted individually. | 7 |
| 19. | Derivation of themes | Descriptive and analytical themes were synthesised in this review. Descriptive themes were generated inductively by comparing similarities and differences of codes between studies. A hierarchical tree structure was made for each descriptive theme. A new theme was created when deemed necessary. Analytical codes were generated by reviewing and reinterpreting the descriptive themes to answer the research question. | 8 |
| 20. | Quotations | The quotation examples are presented in Appendices S5 and S6 to illustrate descriptive themes. The quotations were from participants of the original studies. | S5 Appendix, S6 Appendix |
| 21. | Synthesis output | The self-care needs among international travellers were Self-Care Empowerment, Mutual Understanding, Healthcare Challenges and Opportunities, Preventive Self-Care, and Facilitated Self-Care. | 23 |
