# Supplementary material for: Self-care needs among international migrants and travellers: A systematic review and meta-synthesis
Source: PLoS One. 2026 Mar 10;21(3):e0344437. doi: 10.1371/journal.pone.0344437 (PMC12974874; doi:10.1371/journal.pone.0344437)
Supplement: S3 Appendix — (DOCX) [file pone.0344437.s003.docx]

**S3 Appendix. Search strategies**

**Review title: Self-care needs among international migrants and travellers: A systematic review and meta-synthesis**

PROSPERO register: CRD42022372693

Table A. Search strategy on EMBASE via Ovid

| **No** | **Keywords and Boolean operator** |
| --- | --- |
| 1 | self care.mp. or exp self care/ |
| 2 | self medication.mp. or exp self medication/ |
| 3 | self treatment.mp. |
| 4 | self diagnosis.mp. |
| 5 | self prescription.mp. |
| 6 | self management.mp. |
| 7 | exp non prescription drug/ or non prescription medicine.mp. |
| 8 | over-the-counter.mp. |
| 9 | drug seeking behavior.mp. or exp drug seeking behavior/ |
| 10 | information seeking behavior.mp. or exp information seeking/ |
| 11 | travel.mp. or exp travel/ |
| 12 | traveler.mp. |
| 13 | traveller.mp. |
| 14 | travel related illness.mp. or exp travel related disease/ |
| 15 | international student.mp. |
| 16 | tourist.mp. or exp tourism/ |
| 17 | medical tourism.mp. or exp medical tourism/ |
| 18 | business traveller.mp. |
| 19 | business traveler.mp. |
| 20 | exp migrant/ or exp emigrant/ or exp immigrant/ or exp migrant worker/ |
| 21 | asylum seeker.mp. or exp refugee/ or exp asylum seeker/ |
| 22 | 1 or 2 or 3 or 4 or 5 or 6 or 7 or 8 or 9 or 10 |
| 23 | 11 or 12 or 13 or 15 or 16 or 18 or 19 |
| 24 | 17 or 23 |
| 25 | 14 and 22 |
| 26 | 22 and 23 |
| 27 | 14 and 26 |
| 28 | 11 or 12 or 13 or 14 or 15 or 16 or 18 or 19 |
| 29 | 17 or 28 |
| 30 | 22 and 28 |
| 31 | 22 and 29 |

Table B. Search strategy on International Pharmaceutical Abstract via Ovid

| **No** | **Keywords and Boolean operator** |
| --- | --- |
| 1 | self care.mp. [mp=title, subject heading word, registry word, abstract, trade name/generic name] |
| 2 | self medication.mp. [mp=title, subject heading word, registry word, abstract, trade name/generic name] |
| 3 | self treatment.mp. [mp=title, subject heading word, registry word, abstract, trade name/generic name] |
| 4 | self diagnosis.mp. [mp=title, subject heading word, registry word, abstract, trade name/generic name] |
| 5 | self prescription.mp. [mp=title, subject heading word, registry word, abstract, trade name/generic name] |
| 6 | self management.mp. [mp=title, subject heading word, registry word, abstract, trade name/generic name] |
| 7 | non prescription medicine.mp. [mp=title, subject heading word, registry word, abstract, trade name/generic name] |
| 8 | nonprescription drug*.mp. [mp=title, subject heading word, registry word, abstract, trade name/generic name] |
| 9 | over-the-counter.mp. [mp=title, subject heading word, registry word, abstract, trade name/generic name] |
| 10 | drug seeking behavior.mp. [mp=title, subject heading word, registry word, abstract, trade name/generic name] |
| 11 | information-seeking behavior.mp. [mp=title, subject heading word, registry word, abstract, trade name/generic name] |
| 12 | travel*.mp. [mp=title, subject heading word, registry word, abstract, trade name/generic name] |
| 13 | touris*.mp. [mp=title, subject heading word, registry word, abstract, trade name/generic name] |
| 14 | business travel*.mp. [mp=title, subject heading word, registry word, abstract, trade name/generic name] |
| 15 | travel related illness.mp. [mp=title, subject heading word, registry word, abstract, trade name/generic name] |
| 16 | international student.mp. [mp=title, subject heading word, registry word, abstract, trade name/generic name] |
| 17 | migrant*.mp. [mp=title, subject heading word, registry word, abstract, trade name/generic name] |
| 18 | immigra*.mp. [mp=title, subject heading word, registry word, abstract, trade name/generic name] |
| 19 | asylum seeker.mp. [mp=title, subject heading word, registry word, abstract, trade name/generic name] |
| 20 | refugee.mp. [mp=title, subject heading word, registry word, abstract, trade name/generic name] |
| 21 | medical touris*.mp. [mp=title, subject heading word, registry word, abstract, trade name/generic name] |
| 22 | 1 or 2 or 3 or 4 or 5 or 6 or 7 or 8 or 9 or 10 or 11 |
| 23 | 12 or 13 or 14 or 15 or 16 |
| 24 | 21 or 23 |
| 25 | 17 or 18 or 19 or 20 or 24 |
| 26 | 22 and 23 |
| 27 | 22 and 24 |
| 28 | 22 and 25 |

Table C. Search strategy on MEDLINE via Ovid

| **No** | **Keywords and Boolean operator** |
| --- | --- |
| 1 | exp Self Care/ or self care.mp. |
| 2 | exp Self Medication/ or self medication.mp. |
| 3 | self treatment.mp. |
| 4 | self diagnosis.mp. |
| 5 | self prescription.mp. |
| 6 | self management.mp. or exp Self-Management/ |
| 7 | exp Nonprescription Drugs/ or non prescription medicine.mp. |
| 8 | over-the-counter.mp. |
| 9 | drug seeking behavior.mp. or exp Drug-Seeking Behavior/ |
| 10 | information seeking behavior.mp. or exp Information Seeking Behavior/ |
| 11 | exp Travel/ or travel.mp. |
| 12 | travel related illness.mp. or Travel-Related Illness/ |
| 13 | traveler.mp. |
| 14 | traveller.mp. |
| 15 | exp International Educational Exchange/ or international student.mp. |
| 16 | migrant.mp. or exp "Transients and Migrants"/ |
| 17 | immigrant.mp. or exp "Emigrants and Immigrants"/ |
| 18 | asylum seeker.mp. or exp Refugees/ |
| 19 | 1 or 2 or 3 |
| 20 | 4 or 5 or 6 |
| 21 | 7 or 8 |
| 22 | 9 or 10 |
| 23 | 11 or 12 or 13 or 14 |
| 24 | 16 or 17 |
| 25 | 19 and 23 |
| 26 | 19 or 20 or 21 or 22 |
| 27 | 23 and 26 |
| 28 | 15 or 18 or 23 or 24 |
| 29 | 19 and 28 |
| 30 | 26 and 28 |
| 31 | 1 or 2 or 3 or 4 or 5 or 6 or 7 or 8 or 9 or 10 |
| 32 | 23 and 31 |
| 33 | tourist.mp. |
| 34 | exp Tourism/ or exp Medical Tourism/ or tourism.mp. |
| 35 | 31 or 33 or 34 |
| 36 | 23 and 35 |
| 37 | 36 not 32 |
| 38 | 23 or 33 or 34 |
| 39 | 31 and 38 |
| 40 | 16 or 38 |
| 41 | 31 and 40 |

Table D. Search strategy on APA PsycINFO via Ovid

| **No** | **Keywords and Boolean operator** |
| --- | --- |
| 1 | self care.mp. or exp Self-Care/ |
| 2 | exp Self-Medication/ or self medication.mp. |
| 3 | self treatment.mp. |
| 4 | self diagnosis.mp. |
| 5 | self prescription.mp. |
| 6 | self management.mp. or exp Self-Management/ |
| 7 | exp Nonprescription Drugs/ or non prescription medicine.mp. |
| 8 | over-the-counter.mp. |
| 9 | drug seeking behavior.mp. |
| 10 | exp Information Seeking/ or information seeking behavior.mp. |
| 11 | tourist.mp. |
| 12 | travel?er.mp. |
| 13 | business travel?er.mp. |
| 14 | tourism.mp. |
| 15 | tourism.mp. or exp Tourism/ |
| 16 | exp Traveling/ or travel.mp. |
| 17 | international student.mp. or exp International Students/ |
| 18 | medical tourism.mp. |
| 19 | exp Migrant Workers/ or exp Migrant Farm Workers/ or migrant.mp. |
| 20 | sojourner.mp. |
| 21 | exp Study Abroad/ |
| 22 | backpacker.mp. |
| 23 | digital nomad.mp. |
| 24 | travel-related disease.mp. |
| 25 | 1 or 2 or 3 or 4 or 5 or 6 or 7 or 8 or 9 or 10 |
| 26 | 11 or 12 or 13 or 14 or 15 or 16 or 17 or 18 or 19 or 20 or 21 or 22 or 23 or 24 |
| 27 | 25 and 26 |

Table E. Search strategy on CINAHL via EBSCO

| **Database** | **Search terms** |
| --- | --- |
| CINAHL | (((MM "Self Medication") OR "self medication or self-medication" OR (MM "Self-Care: Non-Parenteral Medication (Iowa NOC)") OR (MM "Self-Care: Parenteral Medication (Iowa NOC)")) OR ((MM "Self Care+") OR "self care or self-care") OR ("self treatment or self-treatment") OR "self-treatment" OR ((MM "Self-Diagnosis+") OR "self diagnosis") OR ("self prescription") OR ((MM "Self-Management") OR "self management or self-management") OR ((MM "Drugs, Non-Prescription") OR "non prescription drugs") OR ("over the counter") OR ((MM "Drug-Seeking Behavior") OR (MM "Information Seeking Behavior") OR "drug seeking behavior"))  AND  (((MM "Medical Tourism") OR "medical tourism") OR (("travelers" OR (MM "Travel+")) OR ("travel related illness") OR "tourist" OR ((MM "Tourism+") OR "tourism") OR ("business travel") OR ("business traveler") OR ("international students" OR (MM "Students, Foreign")))) |
