# Supplementary material for: Self-care needs among international migrants and travellers: A systematic review and meta-synthesis
Source: PLoS One. 2026 Mar 10;21(3):e0344437. doi: 10.1371/journal.pone.0344437 (PMC12974874; doi:10.1371/journal.pone.0344437)
Supplement: S4 Appendix — (DOCX) [file pone.0344437.s004.docx]

**S4 Appendix. Quality assessment of studies**

| **Author(s) (Year)** | **Rationale** | **Q1** | **Q2** | **Q3** | **Q4** | **Q5** | **Q6** | **Q7** | **Q8** |
| --- | --- | --- | --- | --- | --- | --- | --- | --- | --- |
| Arcury et al. (2006) | Yes | Yes | Yes | Yes | Yes | No | Yes | Yes | Yes |
| Fauk et al. (2022) | Yes | Yes | Yes | Yes | Yes | No | Yes | Yes | Yes |
| Khirikoekkong et al. (2023) | Yes | Yes | Yes | Yes | No | No | Yes | Yes | Yes |
| Kilanowski et al. (2010) | Yes | Yes | Yes | Yes | Yes | No | Yes | Yes | Yes |
| Liew et al. (2020) | Yes | Yes | Yes | No | Yes | No | Yes | Yes | Yes |
| Lin et al. (2016) | Yes | Yes | Yes | Yes | Yes | No | Yes | Yes | Yes |
| Madden et al. (2017) | Yes | Yes | Yes | Yes | Yes | Yes | Yes | Yes | Yes |
| McElfish et al. (2016) | Yes | Yes | Yes | Yes | Yes | No | Yes | Yes | Yes |
| McVea (1997) | Yes | Yes | Yes | Yes | No | No | Yes | Yes | Yes |
| Obach et al. (2024) | Yes | Yes | Yes | Yes | Yes | Yes | Yes | Yes | Yes |
| Parent et al. (2022) | Yes | Yes | Unclear | Yes | Yes | No | Yes | Yes | Yes |
| Porqueddu (2017) | Yes | Yes | Unclear | Yes | Yes | No | Yes | Yes | Yes |
| Shahab et al. (2019) | Yes | Yes | Unclear | Yes | Yes | Yes | Yes | Yes | Yes |
| Tyson et al. (2019) | Yes | Yes | Yes | Yes | Yes | No | Yes | Yes | Yes |
| Vajta et al. (2015) | Yes | Yes | Yes | Yes | Yes | Yes | Yes | Yes | Yes |
| Westerling et al. (2020) | Yes | Yes | Unclear | Yes | Yes | No | Yes | Yes | Yes |
| Yan et al. (2020) | Yes | Yes | Yes | Yes | Yes | Yes | Yes | Yes | Yes |

Q1. Was a qualitative approach appropriate? Q2. Was the sampling strategy appropriate for the approach? Q3. What were the data collection methods? Q4. How were data analysed and how were these checked? Q5. Is the researcher’s position described? Q6. Do the results make sense? Q7. Are the conclusions drawn justified by the results? Q8. Are the findings transferable to other clinical settings?
