# Supplementary material for: Self-care needs among international migrants and travellers: A systematic review and meta-synthesis
Source: PLoS One. 2026 Mar 10;21(3):e0344437. doi: 10.1371/journal.pone.0344437 (PMC12974874; doi:10.1371/journal.pone.0344437)
Supplement: S5 Appendix — (DOCX) [file pone.0344437.s005.docx]

**S5 Appendix. Analytical and descriptive themes**

| **Analytical themes** | **Descriptive themes** | **Examples of supporting statements** | | **Interpretations from authors' findings** |
| --- | --- | --- | --- | --- |
| **Healthcare challenges and opportunities** | **Stigma, legal status, and discrimination** | A | *"Most people who see me think I am Colombian or Venezuelan. Maybe because they have this idea in their mind that a Venezuelan or a Colombian woman, because of what they see on social networks or see elsewhere, that these people are very liberal. The intention to approach and propose something to you is there, but knowing that you, even if you answer kindly and more so when you answer kindly, if you give a hint of kindness, they are going to talk, and they talk to you until you don’t stop them or just walk away, they are still going to be there. You try to be nice to the person, but they get confused most of the time and it’s not right. Because you can just say hello and that’s it, but they want to go beyond that and invite you to something."* -Woman, 24, Ecuador **(Obach, 2024)** | - Some migrant women experienced "sexualisation", leading to unequal treatment and harassment. - Male migrants regarded "pride" as important and living with diabetes was seen as a loss of self-pride, thus creating barriers to self-management and help-seeking. - Stereotypes and stigmas manifested into discrimination and unequal treatment. - Not having a temporary residence permit denied some migrants healthcare access, although this practice had no legal basis, and healthcare access should be universal. - Migrants felt that they experienced unequal treatment at healthcare facilities and they believed because of either their insurance status or ethnicity. |
|  |  | B | *“Even if we arrive the first, we are left for the last [to receive care]; that is called discrimination”* -Man, 21, Venezuela **(Obach, 2024)** |  |
|  |  | C | *"At that time, I brought some medicines [ARV] from here [Indonesia], and after taking them all I didn’t try to access the medicines over there because I was afraid of getting caught by the authorities. I didn’t have documents; I was an illegal migrant worker. I was afraid that if I looked for the medicines, then doctors or nurses might ask for some documents and that would be a problem for me. I could get caught and jailed or deported. Sometimes, authorities such as the police went to check for illegal people [migrant workers] in the plantation areas. I remember some friends of mine and I had to run and hide in the forest a few times because of that."* -Isto **(Fauk, 2022)** |  |
|  | **Accessibility** | A | *“When I ﬁrst arrived in Malaysia, I checked on the internet about HIV clinics so I knew. However, I worked in an oil palm plantation which was far from the city. The distance from the plantation to the city where there was an HIV clinic was very far. The problem was that there was no public transportation in the plantation area, so it was very difﬁcult to get to the city. Besides, I didn’t know anyone or friends or relatives in the city, so at that time I thought if I went to the city then who would help me to get the access to HIV clinic and the [ARV] medicines, and where I should sleep because I believed I couldn’t go back to the plantation area on the same day, there was no transportation. Therefore, for almost three years I worked there I didn’t access the medicines.”* -Kobus, 40 years old **(Fauk, 2022)** | - Migrant farmworkers rely on themselves when sick. - A lack of public transport to HIV care services in the city and having no friends and relatives who can help access the services or where they could stay overnight demotivated migrants. - Living far from a health facility was the reason to stay at home, wait and see, or self-medicate. - Distance and transportation also hindered migrants from accessing food at affordable prices, and to seek for rapid diagnostic tests. - An extended hospital stay raised worries about the need for medical repatriation - Some migrants cannot pass the license driving exam due to limited English reading skills. |
|  |  | B | *“[I] live far away from the clinic and [I] could not come. For that reason, I gave him [child] medicines based on my own understanding [on medicines]. Only if he did not feel better, we would come here [MKT clinic]. If the medicines he took made him better, we would not come here. Because [we live] far away from clinic, for that reason we don’t come. But, if he is not better, we would come [to clinic] with bicycle, we would rent other people’s motorbike, or we would come on foot early in the morning.”* – IDI, male participant **(Khirikoekkong, 2023)** |  |
|  | **Finance and insurance** | A | *“…you have to buy half, half of the amount of food to be able to buy your medication.”* -Woman, P26 **(Tyson, 2019)** | - Migrants with low-earning and unstable jobs faced challenges in affording health services. - Many migrant farmworker mothers cited the high cost of meals. - Having large families and financial pressures led to a preference for cheap, high-dense foods. - Although farmworkers wanted to see a doctor, the lack of insurance prevented them. - Despite the willingness, finding insurance providers for elderly tourists can be problematic. - Some migrants can afford insurance, reducing medical expenses and preventing doctor refusal. - Backpackers spent money on activities rather than medical expenses, despite the insurance. |
|  |  | B | *“I would insure myself. Yeah. And now they won’t give, very few people will give you when you are over 80.. .They don’t want to give it to you at that age. You are too big a risk.”* -P1 **(Liew, 2020)** |  |
|  |  | C | *"Health insurance will be much appreciated because when you go to a hospital with no insurance or money to pay, the doctors won’t see the patient."* **(Lin, 2016)** |  |
|  | **Reliance on health services outside the destination country** | A | *“But here, it’s too expensive to go get medical help because if you go to a consultation with the doctors, they charge you a lot of money. That’s why people prefer to buy creams to treat themselves. ... And sometimes he [doctor] doesn’t even give you what you need because, sometimes, they give you a prescription that you have to go buy at the pharmacy. And if they don’t have that cream at the pharmacy, you return home with nothing. But you have already paid the doctor. ... Yes, if it’s not necessary, you don’t go because if they charge three to four thousand dollars, that’s too much money. And if you get sick suddenly and you still feel like you can put up with it, it’s better to go to Mexico because the medicine is cheaper there.”* -M10:342 **(Arcury, 2006)** | - Some migrants postponed their care until they returned to their countries of origin. - Some migrants travelled to other countries with physicians speaking the language they understand. - Some migrants obtained antibiotics in their countries of origin without a prescription and brought them to the destination country. |
|  |  | B | *"If you go there [Bangkok], they can understand any language you speak. The doctors come from many countries and speak many languages like Arabic, French, Spanish, English, and German."* **(Lin, 2016)** |  |
|  | **Reliance on the destination country's health services** | A | *“So I found it very good to the extent like you know that erm, they had all the notes, everything ready and had what they found and what wasn’t found.”* -P11 **(Liew, 2020)** | - Farmworkers went to a pharmacy to consult the pharmacist about their skin conditions. - Hotel staff referred to a pharmacy for free consultation or to a local hospital. - Seeking treatment from a physician was initiated when self-treatment failed. - Migrants had difficulty booking a GP appointment on short notice. Negative sentiments occurred to doctors' consultations. - Despite some communication barriers, tourists were satisfied with the quality of care of the destination country. |
|  |  | B | *"I went to the pharmacist to get free advice and they told me to go see a doctor."* **(Vajta, 2015)** |  |
|  | **Unregulated resources** | A | *“After I was diagnosed with HIV over there [in Malaysia], I asked a friend of mine to help me access HIV treatment, and he told me that he knew a traditional healer who could give [traditional] medicines. So, we both went to that traditional healer, and I got the medicines from him. I took the medicines from that traditional healer for about a year and didn’t access the medicines [ART] from doctors”.* -Sebas, aged 39 **(Fauk, 2022)** | - Some migrants visited traditional healers in the destination country to obtain “treatment” for HIV. - Male migrants self-treated fever and bought sets of polypharmacy from grocery shops, while some preferred injections by unqualified health workers. - Postmails, flea markets, or Mexican specialty stores were some sources to obtain medicines. - Purchasing medicines inside the forest can be costly in the unregulated market. - Migrants obtained antibiotics online without a prescription. |
|  |  | B | *"Y: There is a grocery store in [a North Carolina town]. They sell Mexican food and stuff. They have it there for you to buy. They don't have it out there on the counter, you have to ask for it. Interviewer: They sell medicines for people to inject? Y: Yes, but you have to ask them for it."* **(McVea, 1997)** |  |
| **Facilitated self-care** | **Adherence to treatment** | A | *“On Saturday, I didn’t have. I left my meds behind. So I missed those two days. But erm, the minute I got back on Sunday, I took my meds. So I missed a day and a half.”* -P2 **(Liew, 2020)** | - Migrants on ARV therapy before migrating had the initial intention of accessing HIV care services. - Missed medication doses could be due to inflexible travel itineraries or negligence. - Side effects (e.g. itching, bad palatability, drowsiness) influenced gold miners to stop the treatment. - Non-adherence to diabetes treatment was because of distrust of medications and side effects. - Advice from health workers motivated gold miners to complete malaria treatment. |
|  |  | B | *“I drink it because they tell me to drink it to feel good. Can’t short my treatment.”* -Sala Bora Mining Camp, Region 8 **(Yan, 2020)** |  |
|  | **Self-care products and medicines** | A | *“I usually bring meds, you know, to do me another two weeks. So if I am going to be traveling, I make sure I have enough meds for two weeks after my travel.”* -P3 **(Liew, 2020)** | - Farmworkers either go to a pharmacy for a consultation and OTC products or use home remedies. - Some migrants self-made their traditional medicines using fresh parts of plants and bought herbal medicines at supermarkets or online instead of ARV due to easier access. - Migrants felt easier access to medication in their country of origin. - For "self-care" with lay injections, migrants used a limited range of medicines. - Young migrants felt easy access to condoms through the health system. - Gold miners can access free malaria kits and share them through donations or transactions. - Elderly travellers brought walking sticks and wearable devices (smartwatches). - Travellers buy medications at travel destinations, and it can be challenging without a prescription. - Travellers stored emergency medications in easy-to-access storage and had an extra supply. |
|  |  | B | *“The strips to check your sugar alone cost…between $30 to $50 for 50 strips…that is the most expensive that there is… but I...buy a box and every three months or two months I check the sugar and later I say, ‘No, I have 100, just walking or exercising it will go down.’”* -Man, P15 **(Tyson, 2019)** |  |
|  |  | C | *“I consumed packaged herbal medicines which I bought from supermarkets. Sometimes I ordered them online, but those are a little bit expensive. They were easier to access and I didn’t need any prescription from a medical doctor to get them. It is not like ARV medicines where everybody has to go through a lot of procedures and tests to be able to access them. So, I didn’t think of accessing ARV medicines. I worked nearby a hospital for more than one year and I knew about HIV care services there, but I didn’t access them. I took herbal medicines for nearly two years and then switched to [ARV] medicines once I came back here [to Indonesia]. I continued the treatment using herbal medicines for six months here [in Indonesia] because I bought a few bottles [while abroad] and brought them with me [to Indonesia]. Once I ﬁnished them all, I restarted the ARV medicines.”* -Primus, aged 28 **(Fauk, 2022)** |  |
|  | **Self-treatment** | A | *“[I learned home remedies from] my grandparents mainly. They would tell me, ‘Look, boil this peel/bark and that is good….’ They just tell me that it works because of the bitterness, that it works because of the bitterness and that is how one is controlled. They have their belief that it is because of the bitterness, they say.”* -Woman, P05 **(Tyson, 2019)** | - Self-treatment for fever was with leftover medicines, polypharmacy packs from grocery shops, or herbal medicines. - Self-treatment with herbal medicines was influenced by familiarity with it in the country of origin. - Migrants were unsure about the effectiveness of herbal or traditional medicines. - Rubbing the body with a soaked towel or showering was used to reduce body temperature. - Beliefs of the superiority of injections drove migrants to resort to lay injections. - Seeking treatment from a physician was initiated when self-treatment failed. |
|  |  | B | *“Actually, I took traditional medicines when I was working overseas because they were easy to get or I could make them by myself. It was not because I thought they were more effective than medical treatment (ARVs). Besides, the medicines from doctors (ARVs) were difﬁcult to ﬁnd in the place where I worked, I didn’t even know where to access them”. -Metak, 45 years old* **(Fauk, 2022)** |  |
| **Mutual understanding** | **Communication and language** | A | *“The thing is I didn’t understand French, and neither did my wife. So we were trying to get information and the nurses actually weren’t very pleasant.”* -P11 **(Liew, 2020)** | - Language barriers reduced migrants' confidence and ability to explain their health problems. - Communication issues occurred in Karen/Burmese-speaking migrants at Thai health facilities. - Migrants hoped doctors could speak their languages to make them free from medical interpreters. - Language barriers with local health workers lead to unpleasant experiences. - Better communication with doctors could improve health. - Successful health education among migrants should include materials in their language. |
|  |  | B | *Marshallese participants discuss that “the language barrier is also a barrier to better health.”* **(McElfish, 2016)** |  |
|  | **Intercultural understanding** | A | *"To understand a disease, it’s more important to understand how a patient feels."* **(Lin, 2016)** | - Travel can broaden travellers' language and cultural horizons. - Some migrants travelled to neighbouring countries with physicians speaking their language. - Misunderstanding patients' cultural backgrounds impeded the patient-doctor relationship. - A few migrants felt that the destination country's approach to medication and promotion of self-care was appropriate. The system prioritises self-care, prevention, and minimal use of drugs. - Regulations that have become more inclusive should have been accompanied by training on intercultural health and migratory steps. - Migrants hoped for the recognition of herbal remedies in the destination country’s health system. |
|  |  | B | *Marshallese participants discuss that “the language barrier is also a barrier to better health.”* **(McElfish, 2016)** |  |
| **Preventive self-care** | **Anxiety and mental health** | A | *“I was anxious beforehand about how I would go, given that I wasn’t as strong as I used to be.”* -P3 **(Liew, 2020)** | - Police interventions caused instability and gold miners to live on the run with fear and anxiety. - Migrants tried to hide from authorities and avoided visiting health facilities, minimising the risks of getting caught, detained, or deported. - Undocumented migrants feared deportation, and this became a barrier to healthcare access. - Miners suffered mentally, physically, and socially due to legal, economic, and social exclusions. - A few migrants believed that controlling anger was important in managing diabetes. - Most travellers with cardiovascular diseases felt uncomfortable when travelling solo. - Some elderly travellers are worried about perceived physical fitness during travel. |
|  |  | B | *“The police don’t let them go to work and they have to live on the run. It’s a difficult situation. They come and they break and they burn everything […] They have no job opportunities, they have no profession and many of them are forced to work on the other side, even illegally”* -Key actor A01 **(Parent, 2020)** |  |
|  | **Agency** | A | *“You have to take care of yourself. The boss is not someone who is going to take you to the clinic, unless you are off. For example, it’s happened to my friends when they got sick here, when they felt really bad, they had to miss a few hours of work or even one day so they can get there on their bicycle because the boss doesn’t take anyone.”* -M28:25 **(Arcury et al., 2006)** | - Migrant farmworkers felt the need to rely on themselves when being sick. - Most travellers with cardiovascular diseases felt uncomfortable when travelling solo. - Young migrants were perceived to a lack of self-care for pregnancy prevention. |
|  |  | B | *“It’s a little bit inhibiting in the sense that erm, you know, if I was to go on my own to.. ..I’ll be a little bit nervous. A little bit nervous. You know you are on your own. You didn’t feel well, not just your heart, but anything, you know. If you are on your own, what would you do, yeah.”* -P4 **(Liew, 2020)** |  |
|  | **Dietary choice and control** | A | *“Erm, there’s so much...you can eat as much as you like, and drink as much as you like. But you have to be controlled you see.”* -P6 **(Liew, 2020)** | - Many migrant farmworker mothers were able to identify foods they thought unhealthy and their influences on wellness. They felt time limit affects the preparation of healthy meals for the family. - A few migrant farmworker mothers mentioned the dietary moderation. - Travellers perceived the importance of maintaining a well-controlled diet while travelling. - Diabetes education and self-management practices alone cannot address issues about affordability and the ability to store and prepare healthy food. - Some migrants contrasted the Western diet with their healthier traditional diet. - Some migrants faced difficulty modifying diets comprising culturally traditional food and dishes. - Some ethnic foods labelled unhealthy by migrants were inevitable in cultural celebrations. |
|  |  | B | *“We eat a lot of things made from corn ﬂour, which, it is bad for us…but...[if you’re] 100% Mexican, that’s just the way food is for us!” (laughs)* **(Kilanowski, 2010)** |  |
|  | **Health risk management** | A | *“… wouldn’t be advisable to go and do any strenuous activities they have never done before like you know. Silly things like that. Put themselves in any danger.”* -P4 **(Liew, 2020)** | - Some travellers would avoid extreme leisure activities due to their heart conditions. - Most elderly travellers prefer to travel with their partner or in a group, which can be life-saving. - Migrants who knew the risks of self-injection changed their practices, i.e. using disposable needles or entirely abandoning them. |
|  |  | B | *"Interviewer: So you used disposable needles all the time? F: Yes, and now with AIDS, we don't do it any other way. Who's not afraid of AIDS? I don't know what it is or, for example, I don't know what symptoms to look for, but I know it is dangerous and people who have it die. So you imagine me playing with someone's life."* **(McVea, 1997)** |  |
|  | **Hygiene practice** | A | *“Yes, there must be [a treatment for fungus]. A person has to change daily and take a bath and shave. And you also have to change your underwear. ...Of course, when you take a bath, you have to scrub your whole foot, your toes, everything in order to get rid of the filth because if you don’t get rid of the filth, that will come back.”* -M26:383 **(Arcury, 2006)** | - Washing and bathing immediately relieved several rashes, especially in cool water with refreshment and soothing effects. - Washing the affected areas thoroughly was recognised by a few farm workers. - Some migrant farmworker mothers were aware of hygiene before cooking food for their families. |
|  |  | B | *“Sometimes we don’t have anything to eat because we get back late [around sundown] and have to prepare food and that takes even more time...But I before I cook I like to shower [to get the dirt and chemicals off].”* **(Kilanowski, 2010)** |  |
|  | **Physical activity** | A | *“Oh I will, I walk more when I be abroad. Yeah I would.. ..walk every morning before breakfast, and I will go for cycle every afternoon.”* -P7 **(Liew, 2020)** | - Some elderly travellers expressed physical limitations stemming from their condition during travel. - For some elderly travellers with cardiovascular diseases, travelling in groups provided opportunities to strengthen existing friendships and increase their physical activities. - Although most participants self-reported integrating exercise into their diabetes management, they faced difficulties due to the physical demands of the jobs, long working hours, unpredictable schedules, and competing priorities. |
|  |  | B | *“When they detected that I had diabetes they told me that I had to walk between 30 to 45 minutes. I would go to the park that is here in [city name]. I walked around it twice, but when I started working there and sometimes we get out late, sometimes we get out early, sometimes we go out, we sometimes work for up to 24 hours; with what energy am I going to go and exercise?"* -Woman, P22 **(Tyson, 2019)** |  |
|  | **Self-awareness** | A | *“I won’t go on travel if I don’t feel well. Because I know people who have gone on travel and died on the plane. So I’m unlikely to go if there’s any hint of the risks of me not being well.”*-P3 **(Liew, 2020)** | - Travellers with cardiovascular diseases expressed an appreciation of personal health and responsibility for ensuring their cardiovascular conditions were well controlled and stable before travel, with trip cancellation possible owing to personal illness. - There was a lack of perceived need to seek pre-travel health advice as no travellers sought it. - Some study participants perceived that young people, in general, perceived HIV as low-risk or that they were not afraid enough. |
|  |  | B | *"I don’t think they see the seriousness of the situation about AIDS because they don’t live it. I think they should at least experience a mild infection for them to be afraid. Because they talk about AIDS, but we don’t take care of ourselves when we are young."* -Woman, 21, Ecuador **(Obach, 2024)** |  |
|  | **Social engagement** | A | *"It would be really helpful if the church can put more emphasis on teaching people how to change their mindset about eating all these bad food."* -Participant 19, male, age 36 **(Shahab, 2019)** | - Some migrants knew local people to whom they sought directions to a traditional healer. - Most travellers prefer to travel with their partner or in a group, which can be life-saving for some. - For some elderly travellers with cardiovascular diseases, travelling in groups provided opportunities to strengthen existing friendships and increase their physical activities. - Some migrants could be helped by local friends as interpreters. - Friends and families settled in the destination country helped new migrants orientate the health system. - Migrants practising lay injection depended on family, relatives, and friends. - Religious leaders could potentially influence behavioural change. - Backpackers who travel in groups will support each other in an unfamiliar environment. |
|  |  | B | *"We did a bus trip around Sicily, there were about 35 or 40 of us. So we ﬂew out, on a bus, traveling around...absolute fantastic. Absolutely fabulous."* -P3 **(Liew, 2020)** |  |
|  | **Community interventions and initiatives** | A | *"And here, there is another phenomenon, talking about the general issue of HIV organisations here in Chile. First, they are, in my opinion, few. Secondly, the supply of services is also limited, compared to other neighbouring countries in the region. So, what this means is that, at least, the community-based organisations - which are the ones that have the most access to the population - are the ones that have the least access to resources, so it is the most paradoxical thing. The ones that have the most contact with the population are the ones that have the least access to resources, so, of course, in the overall picture, what this does is that, uh, we seem to be doing things backwards in terms of civil society and HIV."* -Man, 26, Colombia **(Obach, 2024)** | - Health initiatives bolstered young migrants' agency over their SRH and must go conjointly with promoting community-based migrant organisations. - Community initiatives delivered information and linked the health system and migrants. - Many gold miners found the free malaria kit easy to use and carry and returned for a second kit. - Some shared medications at mining sites implied an incomplete medication course. - Religious leaders could potentially influence behavioural change. - Physicians should explain the rational use of antibiotics despite administration/side effects. |
|  |  | B | *"It would be really helpful if the church can put more emphasis on teaching people how to change their mindset about eating all these bad food."* -Participant 19, male, age 36 **(Shahab, 2019)** |  |
|  | **Knowledge and health literacy** | A | *“Well I like to be told exactly about my condition, and the ins and outs of it, and the dangers of it. And advised as to how to behave in a way that is more beneﬁcial for your condition.”* -P1 **(Liew, 2020)** | - Fever carried different meanings, including endemic malaria, dengue, or simply "sick". - Despite malaria, gold miners cited other health risks and problems without understanding them. - The self-diagnostic kit’s “how-to” was in the video as part of its distribution to gold miners. - Antibiotic information was obtained from a health worker, product leaflets, and the Internet. - Being comprehensively well-informed about their conditions was essential for elderly travellers. - Educating migrants about healthy diets motivated them to modify their dietary habits for diabetes. - Migrants who knew the risks of self-injection changed their practices, i.e. using disposable needles or entirely abandoning them. - Some migrants were thought to have sex education before arriving in the destination country. - Travellers felt they needed to know the health system at their destination countries. - Language barriers worsened the navigation of the health system in the destination countries. - Backpackers sought direction from local sources once their illness needed medical attention. - Settled friends, families, and co-workers helped new migrants orientate the health system. - Migrants’ employers asked them to register with a GP without sufficient support for the process. |
|  |  | B | *"I had made up my mind before I left Indonesia and intended to access the [ARV] medicines, but when I was there [in Thailand] I was not brave enough to look for and meet doctors because I didn’t know their language. I didn’t know how to explain what I was going through. Another concern was that I didn’t even know where to start. For example, here [in Indonesia] the procedure is that you just need to go to a public health centre and the nurse will examine your condition and refer you to the HIV clinic. But I didn’t know the procedure over there, so I didn’t make any efforts to access the [ARV] medicines while I was there”* -Ali, aged 48 **(Fauk, 2022)** |  |
|  |  | C | *Most participants got information about antibiotics from their physician or pharmacist. Turkish migrants often asked pharmacists questions about antibiotics. The manufacturers’ leaflets were an important additional source of information in the migrant group. Younger participants also mentioned the internet as an information source.* (**Westerling, 2020)** |  |

GP: general practitioners, HIV: human immunodeficiency virus, ARV: antiretroviral, OTC: over-the-counter, SRH: sexual and reproductive health
