# Supplementary material for: Self-care needs among international migrants and travellers: A systematic review and meta-synthesis
Source: PLoS One. 2026 Mar 10;21(3):e0344437. doi: 10.1371/journal.pone.0344437 (PMC12974874; doi:10.1371/journal.pone.0344437)

**S6 Appendix. Codebook examples and thematic tree**

**Table A. Codebook examples**

| **Analytical theme** | **Descriptive theme** | **Definition** | **Deidentified quote from the original study** |
| --- | --- | --- | --- |
| Healthcare challenges and opportunities | Distance and transport | The degree to which individuals are facilitated in their ability to gain entry to and to receive care and services from the health care system, including geographic, transportational, and financial factors | “That time when I had malaria, [I] used to take Artecom [malaria drug], depends if you feel you know the treatment, you have the symptoms, take Artecom. That time I didn’t take a smear because the interior was far, no hospital.”  “The stores are far away and the food is expensive and...there is no ride to go to the store. We don’t all have cars and there is no bus out here and it’s too far to walk. Most of us don’t know how to drive.” |
|  | Stigma, legal status, and discrimination | Social stereotyping, illegal/undocumented status, or unjust/prejudicial treatments that hinder individuals from accessing healthcare | “Sometimes I feel like they [GP] are saying, ‘why are you coming to see me?”  “Even if we arrive the first, we are left for the last [to receive care]; that is called discrimination” |
|  | Unregulated resources | Informal or loosely regulated business offering services and products needed by international travellers to perform self-care | “[…] I asked a friend of mine to help me access HIV treatment, and he told me that he knew a traditional healer who could give [traditional] medicines. […] I took the medicines from that traditional healer for about a year and didn’t access the medicines [ART] from doctors”  “A lot of people selling medicines and a lot of people buying taking and injecting without understanding. They do not go to hospital/clinic.” |
| Preventive self-care | Dietary choice and control | Control and choice over dietary patterns, which have been found to be important in reducing disease risk | “[…] you can eat as much as you like, and drink as much as you like. But you have to be controlled you see.”  “I try to eat healthy food, [but] sometimes I eat the food that I shouldn’t eat with a lot of grease.” |
|  | Physical activity | Bodily movement produced by skeletal muscles requires energy expenditure, e.g. walking, exercise | “Oh I will, I walk more when I be abroad. Yeah I would.. ..walk every morning before breakfast, and I will go for cycle every afternoon.”  “[…] I would go to the park that is here in [city name]. I walked around it twice, but when I started working there and sometimes we get out late, sometimes we get out early, sometimes we go out, we sometimes work for up to 24 hours; with what energy am I going to go and exercise?" |
|  | Social engagement | Any group or communal activities that trigger and motivate someone to act towards a healthy lifestyle | “[…] I knew some local people, so I asked them about traditional healers and they told me about a traditional healer nearby; he was the one who gave me the traditional medicines. I felt ﬁne, so I didn’t think of taking ARV medicines."  “Just by chatting to other people and the longer you stay then you understand better.” |

GP: general practitioners, ART: anti-retroviral therapy

**Figure A. Thematic tree**


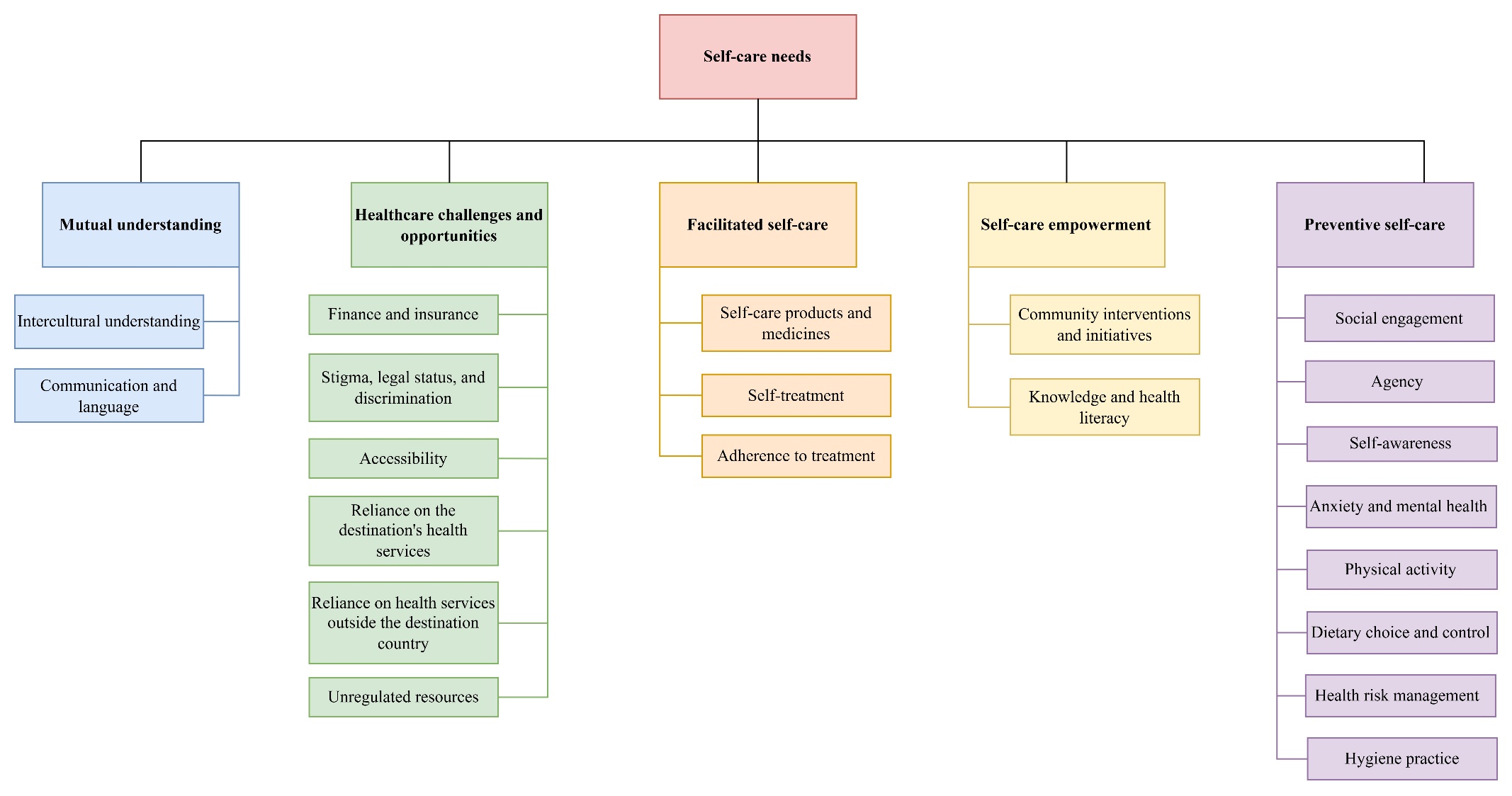

Supplement: S6 Appendix — (DOCX) [file pone.0344437.s006.docx]
