# Supplementary material for: Self-care needs among international migrants and travellers: A systematic review and meta-synthesis
Source: PLoS One. 2026 Mar 10;21(3):e0344437. doi: 10.1371/journal.pone.0344437 (PMC12974874; doi:10.1371/journal.pone.0344437)
Supplement: Attachment — Submitted filename: Response to Editor and Reviewers.docx [file pone.0344437.s008.docx]

# **Responses to Editor and Reviewers**

# Additional Editor Comments:

| **Comment** | **Response** | **Line number** |
| --- | --- | --- |
| Overall, the study addresses a valuable topic but needs clearer framing and stronger methodological justification. You need to thoroughly revise the reviewers' comments to enhance the quality of the manuscript. | Thank you for your appreciation. We have addressed all comments from the reviewers and yours. We have revised the manuscript accordingly. |  |
| Besides reviewers' comments please clarify the distinction between key concepts such as self-care, and self-management. | Thank you for raising this issue. We have differentiated self-care from other concepts, such as self-management.  The manuscript’s Introduction now reads:  “Self-care differs from related constructs, such as self-management (focused on managing chronic diseases), self-efficacy (confidence in performing tasks), and empowerment (capacity building). This review adopted the WHO definition of self-care as “the ability of individuals, families and communities to promote health, prevent disease, and maintain health and to cope with illness and disability with or without the support of a health-care provider”. We define self-care needs as factors that support an individual in benefiting from self-care practices.” | 72-78 |
| The quality appraisal needs to mention all the criteria that were assessed. | Thank you for raising this point. We have added this text below to the Methods section:  “Eligible articles were assessed for quality using the CEBM Critical Appraisal of Qualitative Studies sheet, which consists of eight questions with “Yes”, “No”, or “Unclear” response options. The questions included the study rationale, the appropriateness of a qualitative approach, the sampling strategy, data collection methods, data analysis, the researcher’s position, whether the results made sense, a result-justified conclusion, and transferability of findings.” | 136-139 |
| The discussion needs better interpretations and not just repeating of the findings of the included studies. | Thank you for your suggestion. We have revised the Discussion section to expand our interpretations beyond repeating the findings. For example, we have added this paragraph explaining two key considerations for the Self-Care Matrix framework.  “The SCM was a suitable framework by which we could conduct our analysis. However, we identified two key issues for consideration in the SCM as a sensitising concept in our analysis. First, we identified overlapping and interconnected terms, such as ‘knowledge’ and ‘health literacy’, and ‘mental wellbeing’, ‘self-awareness’, and ‘agency’, which were combined to form an SCM domain or theme. While the use of overlapping concepts offers advantages, such as providing a holistic and realistic understanding of human experience, it can lead to conceptual ambiguity, making it difficult to define, distinguish, and measure constructs accurately. This issue may stem from the development process of the SCM, which brought together 32 self-care models, frameworks, concepts, and theories. Accordingly, we suggest refining the framework through expert consensus. Second, our study's findings demonstrate the value of social interaction for self-care. Specifically, social engagement and social capital can enrich the SCM’s Self-Care Activity and Self-Care Context dimensions, respectively.” | 332-343 |

# Reviewer #1:

| **Comment** | **Response** | **Line number** |
| --- | --- | --- |
| Dear Authors,  Thank you for giving the opportunity to read on the self-care needs of international travellers and migrants through a systematic review and meta-synthesis of qualitative studies. I believe its quality can be improved with some considerations and incorporations as listed below. | Dear Reviewer,  Thank you for your detailed and constructive feedback. Your comments have helped us improve the quality of our manuscript. |  |
| The manuscript introduces self-care matrix (SCM) as the mapping framework, but the theoretical integration is only partial. Please clarify how SCM guided your analysis rather than being applied only at the end for thematic alignment. | We agree that the SCM’s function needs clearer articulation. We added an explanation that SCM was used as a sensitising concept during coding and interpretation, not merely for final mapping.  The Abstract section now reads:  “El-Osta’s Self-Care Matrix (SCM) was used as a sensitising concept and to map conceptual similarities of descriptive themes.” | 30-32 |
|  | The manuscript’s Methods now reads:  “During the synthesis process, the SCM served as a sensitising concept (background ideas that shape and influence how the research problem is approached) during coding and interpretation, ensuring theoretical integration beyond the mapping. The SCM constructed self-care in four dimensions using 34 existing theories, frameworks, and models. Each dimension underscores different aspects of self-care, from personal actions to broader systemic and policy influences. The Self-Care Activities dimension focuses on individual activities and capabilities for self-care and includes the Seven Pillars of Self-Care. The Self-Care Behaviour dimension addresses actions that support positive self-care behaviours and lifestyles. The Self-Care Context considers reliance on resources and their utilisation. The Self-Care Environment focuses on drivers and barriers to self-care within the broader fiscal and policy environment, along with cultural and social factors. Codes and descriptive themes were iteratively compared against the SCM dimensions during analysis.” | 173-184 |
| The concepts of ‘self-care’ and ‘self-care needs’ are used interchangeably with ‘self-management’, ‘self-efficacy’, and ‘empowerment’. A stronger conceptual delineation is necessary in the Introduction and Discussion sections to avoid ambiguity. | We revised the sentences in the Introduction to clarify the distinction between these terms. We moved the sentence that defines self-care needs to after the WHO definition of self-care.  The manuscript’s Introduction now reads:  “Self-care differs from related constructs, such as self-management (focused on managing chronic diseases), self-efficacy (confidence in performing tasks), and empowerment (capacity building). This review adopted the WHO definition of self-care as “the ability of individuals, families and communities to promote health, prevent disease, and maintain health and to cope with illness and disability with or without the support of a health-care provider”. We define self-care needs as factors that support an individual in benefiting from self-care practices.” | 72-78 |
| Authors should consider expanding the theoretical background to discuss how migration, transience, and social capital interact to shape self-care practices. | We added two sentences linking Bourdieu’s concept of social capital and its disruption due to mobility in the Introduction.  The manuscript’s Introduction now reads:  “Regardless of the primary purpose of mobility, migration and transience disrupt social capital (the aggregate of resources embedded in social networks) and influence self-care practices. Reduced social capital may limit access to health information and services in the destination, making social engagement important for rebuilding these networks. Moreover, international travellers and migrants need to understand and adapt to the health system in their destination. Tourists, characterised by a short-term visit, may not need access to healthcare as intensively as long-term migrants, except in emergencies.” | 60-66 |
| The Methods section should explicitly explain the distinct populations of migrants and international travelers in health research.  The inclusion and exclusion criteria should explicitly state how temporary migrants (e.g., seasonal workers) differ from permanent migrants in your dataset and how this distinction was managed during synthesis. | Thank you for highlighting this important point. Our analysis did not intend to compare travellers and temporary migrants as separate subgroups; rather, we treated them as a single conceptual category because they share similar social positions as foreigners and adaptation challenges in unfamiliar environments. Both groups face barriers, including limited social capital, language difficulties, cultural differences, and restricted access to healthcare, impacting their self-care practices. This approach allowed us to derive shared knowledge rather than highlight differences.  To clarify this, we revised the Methods section.The manuscript now reads:  “Travellers and temporary migrants were analysed as a single conceptual category because they share similar adaptation challenges in unfamiliar environments, such as limited social capital, social positions, language difficulties, cultural differences, and barriers to healthcare access. The synthesis was to identify and highlight commonalities across populations rather than differences. Permanent migrants were excluded due to their long-term integration into destination health systems, which differs substantially from the temporary context of transient mobility. Refugees and asylum seekers were also excluded because their health needs may be shaped by forced displacement and trauma, requiring distinct analytical frameworks.” | 107-115 |
| The search strategy in Table 1 is excessively detailed for the main text. You can move it to supplementary materials to improve readability. | We merged Table 1 with S3 Appendix Search strategies and adjusted the numbering of other tables accordingly. |  |
| The quality assessment process using the CEBM checklist is mentioned but not fully justified. Clarify why studies with ‘two or more ‘No’ responses’ were excluded and indicate how inter-rater reliability was maintained during screening and coding. | Thank you for raising this point. We acknowledge that the CEBM checklist does not prescribe a specific threshold for exclusion (i.e. the number of 'No' answers). To ensure methodological rigour and transparency, we established a conservative criterion: studies with more than two “No” responses (out of eight) were excluded. The rationale was that a higher number of negative responses indicates substantial limitations, such as an inadequate sampling strategy or unclear data analysis, which could compromise the credibility and transferability of findings. By setting the threshold at two negative responses, we aimed to balance inclusivity with quality assurance, ensuring that included studies met a minimum standard of methodological rigour.  The manuscript’s Methods now reads:  “Eligible articles were assessed for quality using the CEBM Critical Appraisal of Qualitative Studies sheet, which consists of eight questions with “Yes”, “No”, or “Unclear” response options. The questions included the study rationale, the appropriateness of a qualitative approach, the sampling strategy, data collection methods, data analysis, the researcher’s position, whether the results made sense, a result-justified conclusion, and transferability of findings. The CEBM checklist does not specify exclusion thresholds; therefore, we adopted a conservative criterion, excluding studies with more than two ‘No’ responses to maintain methodological rigour. This pragmatic threshold was chosen because multiple negative responses indicate significant limitations in design or reporting. Researchers ANWP and JC independently assessed each study, and discrepancies were resolved through consensus with Researcher CS. Inter-rater reliability was supported by calibration exercises and documented decision logs.” | 134-145 |
|  | The manuscript’s Results now reads:  “When assessed with the CEBM checklist, common risks for quality were the reporting of the researcher’s positionality (S4 Appendix). However, all identified studies met the quality threshold for meta-synthesis and were included for analysis.” | 219-221 |
| The analytical themes are somewhat descriptive. | Thank you for raising this point. The analytical synthesis process followed Thomas and Harden’s thematic synthesis. In their method, the step of generating analytical themes requires “going beyond” the content of the original studies to produce higher-level interpretations. Thomas and Harden noted that this step is interpretative and is “potentially the most controversial” because it depends on the researchers’ judgment and insights rather than a prescriptive formula. In our study, the analytical themes were generated to synthesise the commonalities across studies by conceptually connecting the descriptive findings.  See our response to Reviewer 2, Comments 2, 3, and 4 for additional detail on the method. |  |
| Figure 2 (mapping to SCM) lacks sufficient explanation in the text. Provide examples showing why certain themes (e.g., ‘social engagement’) did not converge and what this implies for the SCM framework’s completeness. | Thank you for raising this point.  We added the text below to the Results:  “Social Engagement did not converge with the Seven Pillars of Self-Care, which are part of the SCM’s Self-Care Activities dimension. This divergence is because it represents a relational determinant with social networks, i.e. social capital as a meta-contextual factor, rather than a discrete self-care activity.” | 237-240 |
|  | Regarding the implications for the SCM, we have proposed considering social engagement as a component of self-care in the Discussion section.  “We propose adding social engagement as a self-care component for international travel. People have social capital to support health outcomes in a familiar environment. Social capital can be understood as the value of the individuals’ or groups’ social networks. Social capital may decrease when moving to a new, unfamiliar environment. Social engagement can build social capital. “ | 244-249 |
| Several descriptive themes overlap (e.g., ‘health literacy’, ‘knowledge’, and ‘mutual understanding’). | Thank you for bringing this important point to our attention. We acknowledge that several descriptive themes overlap. To clarify, as we used the SCM as a sensitising concept in the analysis, we borrowed their terms to name our descriptive themes, for example, ‘knowledge and health literacy’. This was our challenge when generating codes and mapping the themes to the SCM dimensions.  We added the text below in the Discussion section:  “The SCM was a suitable framework by which we could conduct our analysis. However, we identified two key issues for consideration in the SCM as a sensitising concept in our analysis. First, we identified overlapping and interconnected terms, such as ‘knowledge’ and ‘health literacy’, and ‘mental wellbeing’, ‘self-awareness’, and ‘agency’, which were combined to form an SCM domain or theme . While the use of overlapping concepts offers advantages, such as providing a holistic and realistic understanding of human experience, it can lead to conceptual ambiguity, making it difficult to define, distinguish, and measure constructs accurately. This issue may stem from the development process of the SCM, which brought together 32 self-care models, frameworks, concepts, and theories. Accordingly, we suggest refining the framework through expert consensus. Second, our study's findings demonstrate the value of social interaction for self-care. Specifically, social engagement and social capital can enrich the SCM’s Self-Care Activity and Self-Care Context dimensions, respectively.” | 332-343 |
| The discussion somewhat restates findings rather than critically analysing them in light of prior research on global mobility, health inequalities, and self-care frameworks. You can discuss the role of digital health tools, remote consultations, or mobile health applications for migrants and travellers as emerging enablers of self-care. Clarify how the study contributes new insights to WHO’s self-care agenda or global migration health discourse. | Thank you for your suggestions.  We added this text to the Discussion:  “Our findings extend current discourse on global migration health by highlighting how mobility intersects with self-care practices in ways that are increasingly mediated by digital technologies. Emerging evidence suggests that mobile health applications, remote consultations, and digital health literacy interventions can serve as critical enablers of self-care for migrants and travellers, mitigating barriers to continuity of care and access to health information. This aligns with WHO’s self-care agenda, which positions digital tools as central to empowering individuals. By situating our results within this context, we contribute novel insights into how self-care strategies adapt under conditions of global mobility, offering implications for inclusive digital health design and equity-focused policy development.” | 370-379 |
| Tables (e.g., Table 4) run several pages. Consider summarizing the major analytical findings and moving extended quotations to supplementary files. | Thank you for raising this point. We revised the table by describing the analytical findings. We moved the table with long quotations to the S5 Appendix. |  |
| Figures 1 and 2 need higher resolution and clearer legends. | We increased the resolution of Figures 1 and 2, and provided clearer legends for Figure 2. |  |
| References require formatting consistency throughout as per the journal guidelines. | We identified inconsistent references and reformatted them accordingly. |  |
| The paper at this stage requires substantial revisions in (1) strengthening conceptual clarity around ‘self-care needs’, (2) improving methodological transparency and synthesis rigor, and (3) deepening the interpretive discussion linking findings to theory and practice. Addressing these issues will significantly strengthen the study’s credibility and relevance. I recommend the author to revise the manuscript with the above concerns before making a resubmission to this journal.  I hope you will consider and incorporate the comments to improve the quality of the work and further make it publication ready. Best wishes. | We have addressed all points by strengthening conceptual definitions, clarifying methodology, and deepening theoretical interpretation, predominantly in response to the insightful comments provided by all reviewers. These changes enhance the manuscript’s rigour and relevance. |  |

# Reviewer #2:

| **Comment** | **Response** | **Line number** |
| --- | --- | --- |
| Thank you for your efforts in trying to add knowledge to this important field of study. However, there are some pertinent concerns that have to be addressed. | Thank you for your time and efforts to provide constructive feedback. |  |
| 1) The objective and research question of the review are not clearly stated that the synthesis will answer. | Thank you. We agree and revised the Abstract and Introduction to explicitly state the research question and objective.  Now the Abstract section reads:  “The aim of this review was to determine the self-care needs of international travellers and temporary migrants, and to assess how these needs align with existing self-care frameworks.” | 21-23 |
|  | Now the Introduction section reads:  “Therefore, we conducted a systematic review and meta-synthesis of qualitative literature to identify self-care needs among international travellers and temporary migrants, and to examine how these needs align with the concept of self-care .” | 91-94 |
| 2) This manuscript is co-authored by many and in the methods section, the audience is interested to know what research method was used and the reasons for choosing that method, the participants and sample size, how they were included or excluded from the study, setting, data collection and analysis methods used, limitations and strengths of the study and the IRB authorization if required. There is a section reserved for the participation of each author, which is not in the methods section. Please revise this section to reflect single team dynamics. | We clarified the rationale for using thematic synthesis, as described by Thomas and Harden (2008), and explained its interpretive and iterative nature. We added details on inclusion and exclusion criteria, sample characteristics, and consensus process. The Institutional Board Review or ethics approval was not required, as this study analysed published data – we now explicitly state this. Author contributions are listed in the designated section according to journal guidelines.  The Methods section now reads:  “We applied the thematic synthesis approach by Thomas and Harden as it provides a clear step-by-step guide and allows integration of qualitative findings across diverse contexts and methodologies while preserving interpretive depths. The approach uses three stages for synthesising the data: line-by-line coding, grouping codes into one or more descriptive themes, and generating analytical themes.” | 149-154 |
|  | and  “Ethics approval was not applicable as this meta-synthesis analysed published data.” | 102-103 |
| 3) This is a meta synthesis literature review. Could you please explain the interpretive and iterative process used in the study to identify themes? | We expanded the description of coding and theme development.  The Methods section now reads:  “Coding was conducted line-by-line for all texts under the original study’s ‘results’ or ‘findings’ section. For multi-methods and mixed-methods articles, the texts under the section that indicates the presentation of qualitative findings were subject to extraction. Codes were grouped into descriptive themes through an iterative process of comparison and refinement. Analytical themes were generated by interpreting relationships among descriptive themes in light of the review question and theoretical frameworks.” | 154-159 |
| 4) How did you assess the quality of the included studies? Were there predetermined criteria? | We clarified that the CEBM checklist was used and explained the exclusion threshold.  The Methods now reads:  “Eligible articles were assessed for quality using the CEBM Critical Appraisal of Qualitative Studies sheet, which consists of eight questions with “Yes”, “No”, or “Unclear” response options. The questions included the study rationale, the appropriateness of a qualitative approach, the sampling strategy, data collection methods, data analysis, the researcher’s position, whether the results made sense, a result-justified conclusion, and transferability of findings. The CEBM checklist does not specify exclusion thresholds; therefore, we adopted a conservative criterion, excluding studies with more than two ‘No’ responses to maintain methodological rigour. This pragmatic threshold was chosen because multiple negative responses indicate significant limitations in design or reporting. Researchers ANWP and JC independently assessed each study, and discrepancies were resolved through consensus with Researcher CS. Inter-rater reliability was supported by calibration exercises and documented decision logs.” | 134-145 |
| 5) In a layman's term, what is the novel knowledge or insights generated by this study? | We added a plain-language summary in the Conclusion.  “Our study reveals that when people travel or migrate, they lose social connections that contribute to their overall well-being. Strengthening established social ties and the development of new social ties are key to effective self-care.” | 438-440 |

# Reviewer #3:

| **Comment** | **Response** | **Line number** |
| --- | --- | --- |
| The study focuses on the self-care needs of “traveller” and “migrant” groups with short- or medium-term international mobility, using a systematic review and thematic meta-synthesis of qualitative studies. Searches were conducted in MEDLINE, Embase, IPA, PsycINFO, and CINAHL without date or language restrictions, resulting in the inclusion of 17 studies (n=769). The authors followed ENTREQ and PRISMA reporting standards, used the CEBM qualitative checklist for quality appraisal, applied the three-stage Thomas & Harden synthesis approach, and mapped findings onto the Self-Care Matrix (SCM), identifying five analytical themes. The theme of “social participation” not fully aligning with SCM’s “Seven Pillars” constitutes an important theoretical finding. Overall, the topic is timely, the methodological framework is appropriate, and the findings are meaningful for policy and practice; however, the sample’s overwhelming focus on “migrants,” the conceptual distinction between “traveller” and “migrant,” and certain methodological details require clarification. | Thank you for your observation and constructive comments. |  |
| Only two of the 17 studies focused directly on “travellers” (tourists or backpackers); the remaining studies concentrated on “migrants,” mostly from low-income or context-specific backgrounds. This imbalance limits the transferability of results to both populations mentioned in the title. Therefore, the title, objectives, and discussion should be reframed more transparently and equitably to reflect the actual scope. | Thank you for raising this point. We revised the title, objectives, and discussion to reflect the sample that is predominantly migrant-dominant.  The Title now reads:  “Self-care needs among international migrants and travellers: A systematic review and meta-synthesis”  We added these sentences to the Discussion:  “This review primarily synthesises evidence from transient migrants, with limited data on short-term travellers. Findings should be interpreted with this scope in mind.” | 1-2  327-328 |
| The database selection is appropriate; the update date (30 October 2024) is provided, and a PRISMA flow diagram is reported. However, since no grey literature, projects, or reports were included, publication bias in qualitative studies should be discussed. | Thank you for pointing this out.  The Limitations section now reads:  “Fifthly, excluding grey literature may introduce publication bias, as unpublished qualitative studies could provide additional perspectives. Also, including only qualitative components in mixed-method studies may introduce selection bias. To address potential publication and selection bias, we conducted a sensitivity check by excluding three borderline studies with two ‘No’ responses in the quality assessment. The five analytical themes remained unchanged, with only one descriptive theme (i.e. ‘Health Risk Management’) disappearing, suggesting that the synthesis was sufficiently robust to this exclusion.”  If the editor wishes, we can add the detailed results of this analysis as a supplementary file. | 427-434 |
| The inclusion and exclusion criteria are clearly described; however, the exclusion of “refugee/asylum seeker” groups requires further ethical, sociopolitical, and experiential justification. While it is understandable that forced migrants may have distinct self-care patterns, the impact of excluding them based on the “transient” criterion should be explicitly discussed. | Thank you for raising this issue.  The Methods section now reads:  “Refugees and asylum seekers were also excluded because their health needs may be shaped by forced displacement and trauma, requiring distinct analytical frameworks.” | 114-115 |
| Using the CEBM qualitative checklist and excluding studies with more than two “No” responses is appropriate. Nevertheless, a detailed summary matrix for each study (e.g., the distribution of “Yes/No/Unclear” answers per criterion) should be added as an appendix. | Thank you for your suggestion. We moved Table 3 to S4 Appendix. |  |
| Researcher positionality (reflexivity) was often underreported; this may require weighting studies in the synthesis according to their methodological transparency. | Thank you for raising this important issue. After conducting the quality assessment using the CEBM checklist, the majority of studies (12 out of 17) indeed lack reflexivity reporting. Therefore, we were in a position where we were unable to conduct a sensitivity analysis by excluding those studies to reassess the final themes. Moreover, due to the qualitative design of the included studies, we cannot perform the weighting that quantitative studies can.  We have acknowledged this important point and included it in the Limitations section.  “Secondly, the majority of included studies (12 out of 17) did not report the researcher’s positionality, preventing us from conducting sensitivity analysis by excluding them to reassess the final themes. The qualitative design of the included studies also prevented us from conducting a weighting analysis.” | 417-420 |
| The use of the Thomas & Harden approach is appropriate; however, the reliability of the coding process (dual coding, consensus procedures, codebook examples, and sample coded excerpts) should be described in more detail. | Thank you for highlighting this important point. We provided codebook examples in the S6 Appendix. Sample coded excerpts were previously presented in Table 4, which has been moved to S5 Appendix, as suggested by another reviewer.  The Methods section now reads:  “Coding reliability was ensured through a multi-step process. Researcher ANWP coded each line of the text data from each article according to its meaning and content and developed the descriptive themes by inductively grouping codes into a hierarchical tree structure based on similarities and differences. A preliminary codebook was developed during pilot coding and refined iteratively as new codes emerged. Analytical themes were generated using descriptive themes to answer the review question. Researcher CS reviewed the descriptive and analytical themes. Discrepancies were discussed in detail to reach a consensus, considering the meaning behind each theme and its relevance to the research question. The descriptive and analytical themes were tabulated along with at least two supporting quotes from the original studies to enhance transparency.” | 160-169 |
| NVivo was mentioned, but no information was given regarding inter-coder agreement or comparative control. | Thank you for bringing this issue to our attention.  The Methods section now reads:  “NVivo 13 (QSR International, lumivero.com, Melbourne, Australia) was used for data management and theme development. Formal inter-coder reliability statistics were not calculated, as thematic synthesis prioritises consensus-building over quantitative agreement. All coding decisions were reviewed collaboratively to ensure consistency.” | 169-172 |
| Including only the qualitative parts of mixed-methods studies is methodologically correct; however, this selective inclusion introduces a potential selection bias that could be tested through a brief sensitivity analysis (e.g., checking whether themes remain stable when borderline cases are excluded). | Thank you for your suggestion. We acknowledge this limitation and conducted a sensitivity check. Specifically, we re-examined themes after excluding two studies with borderline cases (minimal qualitative data). The analytical themes remained stable, indicating robustness. We added this information to the Limitations section.  The Limitations section now reads:  “Fifthly, excluding grey literature may introduce publication bias, as unpublished qualitative studies could provide additional perspectives. Also, including only qualitative components in mixed-method studies may introduce selection bias. To address potential publication and selection bias, we conducted a sensitivity check by excluding three borderline studies with two ‘No’ responses in the quality assessment. The five analytical themes remained unchanged, with only one descriptive theme (i.e. ‘Health Risk Management’) disappearing, suggesting that the synthesis was sufficiently robust to this exclusion.” | 427-434 |
| The convergence logic of “full/partial/none” mapping to the SCM is clear, but the process should be supported by independent dual assessment and description of how disagreements were resolved. | Thank you for raising this point.  The manuscript’s Methods now reads:  “To map the meta-synthesis results with current theory in self-care, the SCM was employed. Researchers ANWP and CS developed a mapping procedure to assess conceptual similarities between the meta-synthesis descriptive themes and the SCM’s dimensions and domains. The relationships were categorised into convergence, partial convergence, and lack of convergence. A relationship was convergent when one or more descriptive themes were similar to a dimension or domain. Partial convergence occurred when a descriptive theme related to more than a single dimension or domain. A lack of convergence occurred when a descriptive theme was unrelated to the SCM dimensions or domains. Researcher ANWP conducted an initial mapping, and Researcher CS reviewed the results. Discussions were made to address disagreements with consensus-building.” | 186-195 |
| The authors should also strengthen the theoretical reasoning for why “social participation” did not align with the Seven Pillars (e.g., by framing social capital as a meta-contextual factor influencing self-care behaviors). | Thank you for raising this point.  The manuscript’s Results now reads:  “Social Engagement did not converge with the Seven Pillars of Self-Care, which are part of the SCM’s Self-Care Activities dimension. This divergence is because it represents a relational determinant with social networks, i.e. social capital as a meta-contextual factor, rather than a discrete self-care activity.” | 236-240 |
| Within the theme “self-care facilitation,” the grouping of product/medicine access, self-treatment, and adherence behaviors is well structured; however, the safety and antibiotic resistance risks associated with unregulated sources (markets, online vendors, traditional healers) should be more critically discussed from ethical and public health perspectives. | Thank you for providing suggestions.  We added this into the Discussion:  “Reliance on unregulated medicine sources due to challenging access to healthcare raises ethical and public health concerns, including antimicrobial resistance. Addressing these risks requires targeted health education and regulatory interventions.” | 398-400 |
| The finding on social capital/participation is one of the study’s most original contributions. It should be more visibly linked to literature on help-seeking, network support, and language/cultural mediation (ideally illustrated through a conceptual model diagram). | Thank you for your comment about our original contribution. We are careful in wishing not to overstate the strength of this original finding. Therefore, we opted not to provide a conceptual figure in this case. Should the editor decide that this is an important addition, we would add this. |  |
| ENTREQ and PRISMA adherence is noted; however, the full PRISMA checklist and all search strategies (for each database) should be presented in the supplementary materials. | We have provided the full PRISMA checklist as S2 Appendix and all search strategies as S3 Appendix. |  |
| Tables describing study contexts and samples (age, gender distribution, migration status, duration of stay) should be standardized as much as possible. | We revised Table 2 (now Table 1) by adding information on age, gender distribution, migration status, and duration of stay, based on the explicit availability of these details in the original studies. |  |
| Terminology should be operationally defined at the beginning of the article and used consistently throughout—particularly the distinctions among “traveller,” “migrant,” “transient,” and “international student.” | Thank you for highlighting this important point. Our analysis did not aim to compare travellers and temporary migrants as separate subgroups; rather, we treated them as a single conceptual category because they share similar social positions as foreigners and adaptation challenges in unfamiliar environments. Both groups face barriers, including limited social capital, language difficulties, cultural differences, and restricted access to healthcare, impacting their self-care practices. This approach allowed us to derive shared knowledge rather than highlight differences. To clarify this, we revised the Methods section.  The manuscript now reads:  “Travellers and temporary migrants were analysed as a single conceptual category because they share similar adaptation challenges in unfamiliar environments, such as limited social capital, social positions, language difficulties, cultural differences, and barriers to healthcare access. The synthesis was to identify and highlight commonalities across these populations rather than differences. Permanent migrants were excluded due to their long-term integration into destination health systems, which differs substantially from the temporary context of transient mobility. Refugees and asylum seekers were also excluded because their health needs may be shaped by forced displacement and trauma, requiring distinct analytical frameworks.” | 107-115 |
| The PROSPERO registration is appropriate. The data availability statement (“All relevant data are within the manuscript and its supporting information files”) is acceptable, but adding a de-identified codebook, thematic tree, sample coded excerpts, and quotation list as a “Supporting File” would enhance transparency and reusability. | Thank you for your comments. We added a de-identified codebook and thematic tree as S6 Appendix. Sample code excerpts with quotation examples were supplied as S5 Appendix. |  |
| The PRISMA figure’s readability should be improved; exclusion reasons should be summarized categorically. | We increased the PRISMA’s figure resolution and categorised the exclusion reasons into ‘Not studied population (n = 56)’, ‘Not a journal article (n = 12)’, ‘Not relevant to the topic (n = 11)’, and ‘Neither qualitative nor mixed methods (n = 9)’. Please see Fig 1. PRISMA flow diagram. |  |
| For Tables 2 and 3, studies listed as “data analysis unspecified” should include footnotes explaining this uncertainty and its potential effect on weighting. | We clarified the ‘unspecified’ as the original authors did not explicitly specify their analytical approach. The caption for Table 2 now explicitly states this information.  Table 3 was moved to the S4 Appendix as suggested by another reviewer. |  |

# Reviewer #4:

| **Comment** | **Response** | **Line number** |
| --- | --- | --- |
| The study presents the results of original research. The authors collected and analyzed original empirical data (systematic publication review databases and survey results). | Thank you for reviewing our manuscript and providing constructive feedback. |  |
| The novelty of the study lies in identifying the five most relevant self-care areas (hygiene and self-care) for international travelers and migrants. However, the need to use the complex methodological apparatus chosen by the authors to identify these areas is questionable. If the originality of the study is ensured solely by an extremely cumbersome apparatus, then the result appears theoretically weak: the research costs outweigh the results and do not withstand Occam's razor. | We appreciate this observation. If we understand the comments correctly, there is a concern raised with repect to the meta-synthesis method itself. Among the available methods for meta-synthesis, we chose Thomas & Harden’s thematic synthesis because it provides a clear step-by-step guide and allows for the integration of qualitative findings across diverse contexts and methodologies, while preserving interpretive depth. The approach was chosen to maintain methodological robustness in a meta-synthesis study.  The Methods section now reads:  “We applied the thematic synthesis approach by Thomas and Harden, as it provides a clear step-by-step guide and allows for the integration of qualitative findings across diverse contexts and methodologies while preserving interpretive depth.” | 149-152 |
| I recommend that the authors more precisely define the limitations of their study in the "Limitations" section and clearly indicate where they achieved a scientific breakthrough (novelty) and what the apparatus used did not allow them to achieve. | Thank you for your suggestion. We have changed the topic sentence of the Limitations section.  “There were five limitations in our study, including the migration status, researcher’s positionality, and research focus of the original studies, the COVID-19 context, and the risks of publication and selection bias.” | 410-412 |
|  | We clarified the novelty in the Discussion and Conclusion by emphasising theoretical integration and practical implications.  The Discussion now reads:  “Our study advances theory by the integration of social capital into the SCM, addressing a gap in travel and migration health literature.” | 330-331 |
|  | The Conclusion now reads:  “This review contributes to self-care theory in the context of international mobility by advancing social capital as a meta-contextual factor and offers practical guidance for policy and digital health interventions.” | 440-443 |
| Furthermore, the "Conclusion" section does not contain general conclusions, as if the authors conducted the study solely to learn that research should be conducted. This is a theoretical oxymoron: they studied at length, but what they learned is unclear. The conclusion needs to be seriously revised and significantly strengthened theoretically. | Thank you for raising this point.  We added these sentences to the Conclusion:  “This study has identified five primary self-care needs: self-care empowerment, mutual understanding of cultures, healthcare challenges and opportunities, preventive self-care, and facilitated self-care. Our study reveals that when people travel or migrate, they lose social connections that contribute to their overall well-being. Strengthening established social ties and the development of new social ties are key to effective self-care. This review contributes to self-care theory in the context of international mobility by advancing social capital as a meta-contextual factor and offers practical guidance for policy and digital health interventions.” | 436-443 |
| 2. Results reported have not been published elsewhere. The authors rely on the analysis of collected material that has not been published before. | Thank you for your acknowledgment. |  |
| 3. Experiments, statistics, and other analyses are performed to a high technical standard and are described in sufficient detail. The authors presented the study's algorithm and the statistics that influenced the results in sufficient detail. However, the authors' preliminary conclusions raise doubts. Overcoming subjective limitations is not an end in itself. Clear criteria for qualitative evaluation are needed. The authors need to propose to the scientific community ways to replicate their results so that the feasibility of achieving them becomes clear. Otherwise, the obtained results cannot be considered scientific. | Regarding clear criteria for qualitative assessment, we used the CEBM checklist to include/exclude original studies. We pragmatically established more than two “No” responses as a cut-off for exclusion because the CEBM checklist does not prescribe a specific threshold.  The manuscript’s Methods now reads:  “The CEBM checklist does not specify exclusion thresholds; therefore, we adopted a conservative criterion, excluding studies with more than two ‘No’ responses to maintain methodological rigour. This pragmatic threshold was chosen as multiple negative responses indicate significant limitations in design or reporting. Researchers ANWP and JC independently assessed each study, and discrepancies were resolved through consensus with Researcher CS. Inter-rater reliability was supported by calibration exercises and documented decision logs.”  Regarding replicability, we have provided a study protocol at PROSPERO, comprehensive search strategies (S3 Appendix), examples of quotes (S5 Appendix), and a codebook excerpt and thematic tree (S6 Appendix). | 136-142 |
| 4. Conclusions are presented in an appropriate fashion and are supported by the data. Some preliminary conclusions appear well-founded and supported by statistics. However, the criteria for consensus among the research team members in formulating a general conclusion on the theoretical value of the analyzed scientific literature are unclear. The final conclusion requires significant revision. | Thank you for your suggestion. We revised the Conclusion by emphasising the original contribution of this review.  The Conclusion now reads:  “This study has identified five primary self-care needs: self-care empowerment, mutual understanding of cultures, healthcare challenges and opportunities, preventive self-care, and facilitated self-care. Our study reveals that when people travel or migrate, they lose social connections that contribute to their overall well-being. Strengthening established social ties and the development of new social ties are key to effective self-care. This review contributes to self-care theory in the context of international mobility by highlighting social engagement as a meta-contextual factor and offers practical guidance for policy and digital health interventions.” | 436-443 |
| 5. The article is presented in an intelligible fashion and is written in standard English. The ratio of professional and everyday vocabulary within the standards of scientific style. The structure of the article reflects the logic of scientific research. The style of link formatting should be adjusted according to the editorial requirements. | If we correctly understand the suggestion, we have reviewed and adjusted the style of link formatting (references) to meet editorial guidelines. |  |
| 6. The research meets all applicable standards for the ethics of experimentation and research integrity. The author does not violate standard standards of experimental ethics and research integrity. | Thank you for your acknowledgement. |  |
| 7. The article adheres to appropriate reporting guidelines and community standards for data availability. The author adheres to relevant reporting guidelines and standards for data accessibility accepted in the scientific community. | Thank you for your acknowledgement. |  |
