# Supplementary material for: Self-care needs among international migrants and travellers: A systematic review and meta-synthesis
Source: PLoS One. 2026 Mar 10;21(3):e0344437. doi: 10.1371/journal.pone.0344437 (PMC12974874; doi:10.1371/journal.pone.0344437)
Supplement: Attachment — Submitted filename: Response to Reviewers.docx [file pone.0344437.s009.docx]

# **Responses to Reviewers**

# Reviewer #2:

| **Comment** | **Response** | **Line number** |
| --- | --- | --- |
| The authors have not adequately addressed the comments referring to portraying the study as teamwork and not singling out parts played by individual researchers in the methods section. Readership is not interested in who did what but would like to know the methodological flow of the study. For example, mentioning that "Researcher ANWP coded each line of the text data..." is irrelevant in the methods section. Please address all similar sentences. There is a section reserved for that. | Thank you for your time and effort to provide constructive feedback. We have singled out parts played by individual researchers in the Methods section as suggested.  The Methods section now reads: |  |
|  | “A literature search was conducted, followed by manual and automated deduplication using Covidence.” | 127-128 |
|  | “Twenty records were selected for initial pilot screening.” | 128 |
|  | “Title and abstract screening of the remaining articles was conducted afterwards, followed by full-text screening.” | 128-129 |
|  | “Discrepancies were discussed until a consensus was reached.” | 130 |
|  | “Each study was independently assessed, and discrepancies were resolved through consensus” | 143-144 |
|  | “Each line of the text data from each article was coded according to its meaning and content, and descriptive themes were developed by inductively grouping codes into a hierarchical tree structure based on similarities and differences.” | 160-163 |
|  | “A mapping procedure was developed to assess conceptual similarities between the meta-synthesis descriptive themes and the SCM’s dimensions and domains.” | 186-187 |

# Reviewer #3:

| **Comment** | **Response** | **Line number** |
| --- | --- | --- |
| The manuscript has now been clearly revised and is suitable for publication.  Most of the previous critiques have been addressed systematically: the “traveller vs. migrant” distinction has been clarified in the title, abstract, and methods section, with both groups defined as sharing a common context of temporary mobility. | Thank you for your acknowledgements. |  |
| The potential bias introduced by excluding grey literature has been acknowledged in the “Limitations” section. | Thank you for your acknowledgements. |  |
| The CEBM quality appraisal table has been provided in detail in the supplementary files (S4 Appendix). | Thank you for your acknowledgements. |  |
| The coding process has been strengthened with two coders, a third reviewer, calibration exercises, and decision logs, substantially enhancing the reliability of the analysis. | Thank you for your acknowledgements. |  |
| The SCM mapping was conducted independently by two researchers and validated through a consensus-building approach. | Thank you for your acknowledgements. |  |
| A “Reflexive Statement” section (pp. 17–18) has been added, outlining the team’s experience. | Thank you for your acknowledgements. |  |
| A sensitivity analysis has been included in the “Limitations” section (pp. 33–34), demonstrating that the analytical themes remained stable. | Thank you for your acknowledgements. |  |
| The PRISMA diagram has been refined, Table 1 has been improved, and sample information is now clearer. | Thank you for your acknowledgements. |  |
